# Cyclic Oligosaccharides as Active Drugs, an Updated Review

**DOI:** 10.3390/ph13100281

**Published:** 2020-09-29

**Authors:** Adrián Matencio, Fabrizio Caldera, Claudio Cecone, José Manuel López-Nicolás, Francesco Trotta

**Affiliations:** 1 Dipartimento di Chimica, Università di Torino, via P. Giuria 7, 10125 Torino, Italy; fabrizio.caldera@unito.it (F.C.); claudio.cecone@unito.it (C.C.); francesco.trotta@unito.it (F.T.); 2 Departamento de Bioquímica y Biología Molecular A, Unidad Docente de Biología, Facultad de Veterinaria, Regional Campus of International Excellence “Campus Mare Nostrum”, Universidad de Murcia, 30100 Espinardo, Murcia, Spain; josemln@um.es

**Keywords:** cyclodextrins, cyclic nigerosyl-1,6-nigerose, treatment, review, drug, intrinsic

## Abstract

There have been many reviews of the cyclic oligosaccharide cyclodextrin (CD) and CD-based materials used for drug delivery, but the capacity of CDs to complex different agents and their own intrinsic properties suggest they might also be considered for use as active drugs, not only as carriers. The aim of this review is to summarize the direct use of CDs as drugs, without using its complexing potential with other substances. The direct application of another oligosaccharide called cyclic nigerosyl-1,6-nigerose (CNN) is also described. The review is divided into lipid-related diseases, aggregation diseases, antiviral and antiparasitic activities, anti-anesthetic agent, function in diet, removal of organic toxins, CDs and collagen, cell differentiation, and finally, their use in contact lenses in which no drug other than CDs are involved. In the case of CNN, its application as a dietary supplement and immunological modulator is explained. Finally, a critical structure–activity explanation is provided.

## 1. Introduction

**Figure 1:**
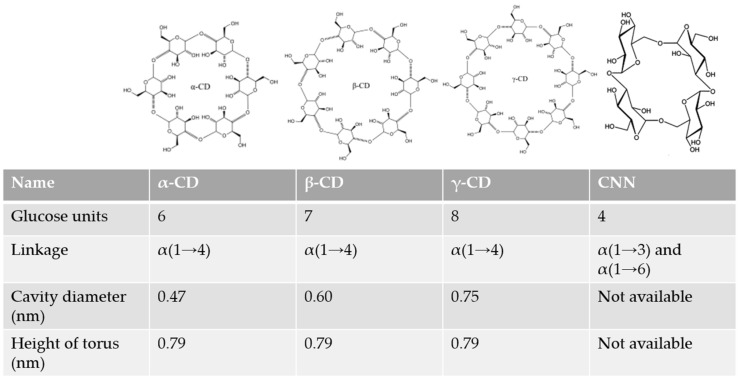
Structure of natural cyclodextrins (CDs) and cyclic nigerosyl-1,6-nigerose (CNN).

Cyclodextrins (CDs, Figure 1) are torus-shaped oligosaccharides made up of α-(1,4)-linked glucose units, obtained by the degradation of starch by the enzyme cyclodextrin glucosyltransferase (CGTAse), which were (CDs) discovered by Antoine Villiers [1]. Although the most common CDs are the natural α, β, and γ-CD forms (which contain six, seven, or eight glucose units, respectively), CDs containing nine and up to nineteen units have also been characterized [2,3], although they are not used because of their tendency to collapse. Recently, even smaller CDs with 3 or 4 glucose units have been synthesized [4].

The CD ring is a conical cylinder of an amphiphilic nature, with a hydrophilic outer layer (formed by the hydroxyl groups) and a lipophilic cavity [5]. Although inorganic and organic salts and neutral molecules can form complexes with CDs [6], it is generally poorly soluble drugs that are complexed with them to create so-called “inclusion complexes” or nanoparticles [7,8,9,10,11,12].

Different chemically obtained derivates (e.g., hydroxylpropyl-β-CD or methyl-β-CD among others) and materials have been seen to possess better capacities, such as complexation efficiency and release accuracy, than natural CDs [13,14,15]. However, only natural CDs are considered to be suitable as food additives at present (E-457, E-458, and E-459, [5]).

Despite the importance of CDs in science, especially as carriers in pharmacy [16,17], their use as active drugs has been less studied: despite the recent reviews [18,19,20] that have focused on this particular application. In addition to CDs, another dietary indigestible cyclic oligosaccharide formed by four D-glucopyranosyl residues linked by alternating α(1→3) and α(1→6) glucosidic linkages was recently found to have intrinsic bioactivity cyclic nigerosyl-1,6-nigerose or cyclotetraglucose (CNN, Figure 1 [21]) The present review will update the most relevant applications mentioned in the review made by Braga et al., 2019, including applications, such as the ability of CDs to combat aggregation diseases, their dietary functions, toxins removal, cell differentiation, and their application in contact lenses. The review aims to provide a general overview of the use of different oligosaccharides as active drugs, rather than as mere drug carriers, summarizing and updating the most relevant applications mentioned in previous reviews and adding new possible uses.

## 2. Pharmacokinetics, Toxicology, and Regulation

### 2.1. Cyclodextrins

Salivary α-amylase can rapidly hydrolyze dextrins, although their rapid transport to the stomach makes such degradation unimportant. Of the three natural CDs, α- and β-CD are essentially stable towards α-amylase, while γ-CD is rapidly digested [16,22]. In the stomach, unspecific pH dependent degradation may occur, and then, in the neutral pH environment found in the small intestine, pancreatic amylase continues the hydrolysis process. While α- and β-CD are mostly digested by bacteria in the colon (α-CD is more slowly digested than β-CD, [16]), γ-CD is almost completely digested in the gastrointestinal tract. Finally, non-digested remains are metabolized by microbiota in the lower section of the digestion system, where they are almost completely degraded. The low bioavailability of CDs and their derivatives (they are not able to pass the intestinal barrier) makes them very safe when administered orally [16,23,24].

Since dextrins can also be administered parentally, their pharmacokinetics has also been studied, leading to monographs such as those included in the European Pharmacopoeia [25]. As regards their pharmacokinetics, dextrins below 15 kDa are almost totally excreted (≥90%) in the urine without any substantial modification. More specifically, the pharmacokinetics of hydroxypropyl-β-CD (HPβ-CD), sulfobutylether β-CD sodium salt and Sugammadex sodium salt has been studied in rats, where they showed a t_1/2_ of 1.9, 1.6, and 1.7 h respectively. More than 90% of the CD was recovered in the urine in 24 h, although the CDs may remain longer in the kidney of affected subjects [16,24,26,27,28,29,30].

In terms of toxicity, most studies refer to the medical uses. A high dose of orally administered CDs can generate diarrhea and caecum enlargement or even affect the bioavailability of some substances; as a consequence of which, the European Commission prepared a guide to help during drug development [31]. On the other hand, the toxicology of HPβ-CD has been better studied due to its classical use as a medical excipient [32], and its degree of substitution [33]. The results showed that the best option for minimizing toxicity would be to have a low degree of substitution (D.S); however, more studies are necessary in this respect.

As regards the use of CDs for supplementing food products, only natural CDs are considered food additives (E-457, E-458, and E-459) and “generally recognized as safe” (GRAS). The recommendation of the Joint FAO/WHO Expert Committee on Food Additives (JECFA) established a maximum level of β-CD in foods of 5 mg/kg/day. On the other hand, for α- and γ-CD there is not an acceptable daily intake (ADI) recommendation due to their favorable toxicological profiles. The European Food Safety Authority (EFSA) recognized that α-CD could be described as dietary fiber, and as suitable for reducing post-prandial glycemic responses [34] due to is competitive inhibition of α-amylase. Generally, GRAS molecules are directly approved for use as excipients (in this case, natural CDs). Moreover, the Food and Drug Administration (FDA) has published a list of inactive pharmaceutical ingredients that can be downloaded (https://www.fda.gov/Drugs/InformationOnDrugs/ucm113978.htm). In this list, the route, dosage form, and maximum concentration is indicated. Additionally, the European Medicines Agency (EMA) has published several reports on the use of different CDs in pharmaceutical products (https://www.ema.europa.eu/en/cyclodextrins). A recent question and answer document about CDs and their uses [31] summarizes information on safety: for example, although about 200 mg/kg/day of CD is generally recommended for oral uses in pharma, this value will depend on the type of CDs used; a dosage of 8000 mg/day, for instance, in the case of HPβ-CD. Another organization, the Japanese Pharmaceutical Codex (JPC), has published monographs about CDs [35].

### 2.2. Cyclic Nigerosyl-1,6-Nigerose or Cyclotetraglucose

In general, cyclic oligosaccharides are “generally recognized as safe” (GRAS) [36]. CNN is a food additive approved by the FDA but no ADI is reported [37]. According to a summary of WHO reporting, several unpublished data about the safety of CNN [38], salivary, or pancreatic α-amylase are not able to degrade CNN in vitro [39]. The in vivo digestibility of CNN was also checked in 35 rats given 100 mg CNN/kg. Approximately 94% of the administered CNN dose was excreted intact in the feces, while the remaining portion (6%) was detected in the gastrointestinal tract undergoing slow degradation by microbiota. No CNN was detected in the blood during the experimental period [39]. Studies about acute toxicity (male and female rats administered a single dose of CNN of 200, 2000, or 5000 mg/kg for 15 days) or short-term toxicity (male and female rats given a mean daily dose of 1568, 2012, and 6333 mg CNN/kg for males and 1799, 3597, and 7270 mg CNN/kg for females) did not reveal signs of toxicity or changes in weight, biochemical parameters, or abnormalities. However, the oral intake of 20 or 30 g not less than 2 h after lunch or dinner had a laxative effect in a human clinical trial [38].

## 3. Cyclodextrins as Active Drugs

Although CDs are mainly used as excipients for carrying different molecules, new applications have been explored, including: (i) to complex metabolites (e.g., cholesterol), (ii) as dietary fibers, (iii) to reduce contaminants, (iv) as antiviral or antiparasitic agents.

### 3.1. Lipid-Related Diseases

In the field of lipid-related diseases, the complexation of cholesterol is the principal application of CDs [40]. However, in this section, we will also look at different targets associated with several diseases.

#### 3.1.1. Niemann-Pick Disease Type C

**Figure 2:**
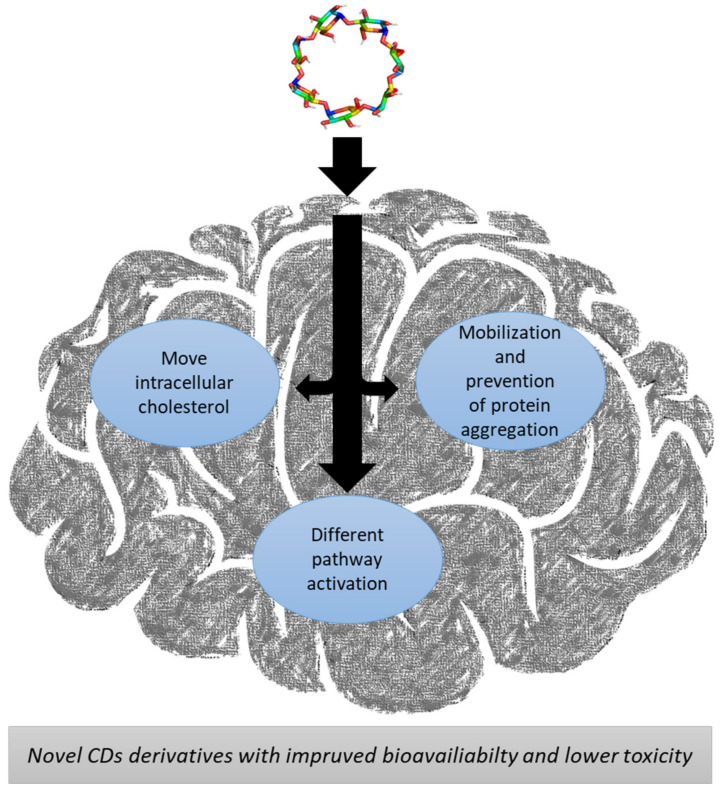
Schematic mechanism of action of CDs in NPC (Niemann Pick Type C), PD (Parkinson’s disease), AD (Alzheimer’s disease), and HD (Huntington’s disease).

Niemann Pick disease Type C (NPC) is a rare recessive disease caused by the mutation of NPC1 and/or NPC2 genes, which change the processing of low-density proteins (LDL) resulting in an accumulation of lipids in the cells [41]. The most promising treatment would seem to use CDs due to their ability to form inclusion complexes (Figure 2) with lipids and to mobilize the deposits [30].

It has been demonstrated that methyl-β-CD (Mβ-CD) and HPβ-CD can reduce cholesterol accumulation [29,42,43]. Although the mechanisms that modulate cholesterol homeostasis are unclear, several hypotheses exist [44]: (i) to control the plasmatic membrane cholesterol, CDs remove the cholesterol from the membrane, which is replaced by intracellular cholesterol; (ii) the CDs enter the cell by pinocytosis, capturing the intracellular cholesterol [45] (it has recently been demonstrated that CDs reach the cytosol and autophagy vesicles [46]); or (iii) the presence of CDs activates an unknown system to remove the cholesterol, as in the results obtained with HPγ-CD (CD unable tp complex cholesterol) suggest [44]. Although treatment with CDs have adverse effects, the blood lipids protect the membrane against injury by CDs; indeed, the treatment with HPγ-CD and HPβ-CD [44] induced the expression of protein-like lysosomal-associated membrane protein 1 (LAMP-1), which is expressed in the lysosomal membrane. It is possible that cholesterol is linked to this protein, thus facilitating its sequestration [47].

However, the principal difficulty of treatment with CD is the molecule’s inability to cross the blood–brain barrier, and while subcutaneous injection can decrease the level of cholesterol in several tissues, an intrathecal injection is needed to have any effect in the brain [48,49]. Indeed, intrathecal injections prevent some adverse effect of CD treatment such as lung toxicity. In 2018, Berry-Kravis et al., studied the neurological function of three patients treated intrathecally with HPβ-CD [50] and observed slight improvement in cognitive ability, mobility, equilibrium, and swallowing.

Finally, different CD monomers are at present being synthetized to optimize treatments, such as *6-O*-maltosyl-β-CD (G2-β-CD) [51], mono-lactose β-CD (Lac-β-CD), and multi-lactose (multi-lac-β-CD) [52] or octa-arginine derivatives [53].

Another possibility is to use polymers, formed by covalent bonds or cyclodextrin-based polyrotaxanes (CDPRX) [54]. As the cavity is covered by the polyrotaxane, the CD cannot take cholesterol of the membranes, reducing its toxicity. Moreover, the structure of CDPRX improves endocytosis [54]. Another interesting polymer is ORX-301, a pH sensitive β-CD-based polymer with a better pharmacokinetics and bioavailability [55].

#### 3.1.2. Atherosclerosis

Atherosclerosis is a vascular disease caused by cholesterol accumulation on the walls of arteries. The inflammatory response starts with the recruitment of macrophages to remove the cholesterol, forming “foam cells” in combination with others such as smooth muscle cells or endothelial cells [56,57]. This excessive cholesterol accumulation modifies the cellular cholesterol pools. The regulation of cholesterol is carried out by ABCA1 (ATP-binding cassette transporter, also known as the cholesterol efflux regulatory protein), ABCG1 (ATP-binding cassette sub-family G member 1), and SR-B1 (scavenger receptor class B type 1) transporters, which remove the cholesterol from the cell membranes towards the extracellular HDL (high-density lipoprotein); in this process, the alteration of any of these transporters may cause atherosclerosis [58].

Another therapy that has been considered is to use CD to form complexes with cholesterol solubilizing the plaques. Although a recent review has been published about this particular application [20], a summary of the most interesting studies is presented below. For example, the potential use of KLEPTOSE^®^ CRYSMEβ (Mβ-CD) was tested in vivo [59] in an apoE-deficient mouse model. The animals were fed with a normal or cholesterol-rich diet, and a vehicle (PBS) or the KLEPTOSE^®^ CRYSMEβ solution was injected. Independently of the diet, the results showed an increase in HDL cholesterol and a decrease in blood triglyceride levels. Moreover, KLEPTOSE^®^ CRYSMEβ also reduced the atherosclerotic plaque by lowering cholesterol levels to a greater extent than the control. In another in vitro study, the effect of several β-CDs and their methylated derivatives on the cholesterol metabolism was tested in cell cultures [60]. Another group recently published their interesting findings concerning atherosclerosis: β-CD (probably Mβ-CD, although this is not specified) was found to be a shuttle of cholesterol at low β-CD concentrations and a sink of cholesterol at high concentrations [61]. This study showed that β-CDs can extract cell membrane cholesterol. This extraction process has been correlated with the strongly decreased expression of ABCA1 and ABCG1 transporters.

The use of the CD derivative HPβ-CD was also evaluated. The CD was able to treat atherosclerosis not only by increasing the efflux of cholesterol [62] but also through macrophage reprogramming [63]. The last authors demonstrated in vivo that the underlying mechanism of action of HPβ-CD involves the liver X receptor (LXR)-mediated signaling pathway; cholesterol efflux was increased as a result of ABCA1 and ABCG1 upregulation, which was corroborated in another recent study where this CD reduced the levels of plasma triglycerides and inflammatory cytokines and also increased the level of plasma HDL-cholesterol. Importantly, the atherosclerotic lesion areas and the macrophage and collagen contents in the atherosclerotic lesions were reduced [64]. Pilely et al., in 2019, discovered that α-CD inhibits cholesterol crystal-induced complement-mediated inflammation better than HPβ-CD. It is even able to solubilize cholesterol crystals [65].

The role of α-CD was also studied in atherosclerosis. As this CD is approved for use as a nutritional supplement, it was thought its oral intake might help treat atherosclerosis by reducing the uptake of cholesterol. However, only a modest reduction in LDL [66] was observed, possibly due to the low bioavailability of α-CD [16]. Nevertheless, in another study α-CD was able not only to reduce atherosclerosis, but also to change the microbiota [67]. It is clear that more studies are needed in this field to clarify the real effect of CDs.

When synthetic CD polymers were evaluated, those with a diameter of ~ 10nm were found to exhibit outstanding pharmacokinetics and plaque targeting efficacy compared with monomeric CD in vivo and showed no ototoxicity [68].

To sum up, the potential effects of cyclodextrins on atherosclerosis progression are [20]:

(i) To inhibit the entry of circulating monocytes into the lesion by inhibiting their adhesion to the endothelium.
(ii) To stimulate cholesterol efflux, and interact with and contribute to the dissolution of cholesterol crystals.
(iii) To decrease the susceptibility of LDL to oxidation.
(iv) To inhibit cholesterol crystal-induced phagocytosis.
(v) To inhibit cholesterol crystal-triggered complement activation.
(vi) To reduce inflammation and production of reactive oxygen species in atherosclerotic lesions.

### 3.2. Aggregation-Related Diseases

#### 3.2.1. Parkinson Disease

Parkinson disease (PD) is caused by α-synuclein protein aggregation and misfolding [69]. It has been reported that CDs (in particular Mβ-CD) have the capacity of complex α-synuclein preventing its aggregation [70]. On the other hand, the essential activity of the transcription factor EB (TFEB) could be activated pharmacologically using HPβ-CD, a master regulator of the autophagy–lysosomal pathway [71], to prevent the accumulation of synuclein aggregates [72] (Figure 2). Although these are promising results, more work is necessary in this field to clearly understand the potential use of CDs.

#### 3.2.2. Alzheimer Disease

Alzheimer disease (AD) results from an accumulation of β-amyloid peptides (AP) in the brain, which is linked to an abnormal cholesterol metabolism [73,74,75]. In this disease, CDs present interesting possibilities; β-CD and HPβ-CD can bind AP directly [76,77,78]. In mouse model, the administration of HPβ-CD reduced β-amyloid (Figure 2) deposits as a result of a reduced APP protein cleavage while upregulating *abca1* and *npc1* gene expressions [79]. As a consequence, alginate or chitosan microparticles containing CDs have been created to target the brain with no toxicity [80] and more effective CD derivatives with dimethylamino aromatic moiety [81].

#### 3.2.3. Huntington’s Disease

Huntington’s disease (HD) is a rare autosomal dominant neurodegenerative disease caused by a mutation within the Huntingtin gene leading to the expansion of a CAG triplet repeat encoding for a polyglutamine (polyQ) tract. This leads to the expression of a mutant form of the Huntingtin protein bearing an expanded polyQ tract and other peptides produced by translational frameshifting or ATG-independent translation. The accumulation of these toxic peptides, through gain- and loss-of-function mechanisms [82,83,84,85], drives the neurodegenerative processes in HD, leading to motor, behavioral, and cognitive dysfunctions generally related to the length of the CAG expansion, with some individual variability [86]. In an interesting study, treatment with β-CD reduced the content of ordered domains of cholesterol at the cell surface, which in turn, protected cells against NMDA(*N*-Methyl-d-aspartate)-mediated excitotoxicity (Figure 2). This is because mutant huntingtin produces the accumulation of cholesterol and alters its cellular distribution, thus contributing to NMDA-mediated excitotoxicity [87].

### 3.3. Antiviral Activity

The capacity to complex cholesterol is the principal mechanism through which CDs can reduce the infectivity of several viruses, as summarized by Braga in 2019 [18]. However, in recent studies novel derivates, which are able to directly block some parts of the virus have been prepared.

#### 3.3.1. Influenza Virus

**Figure 3:**
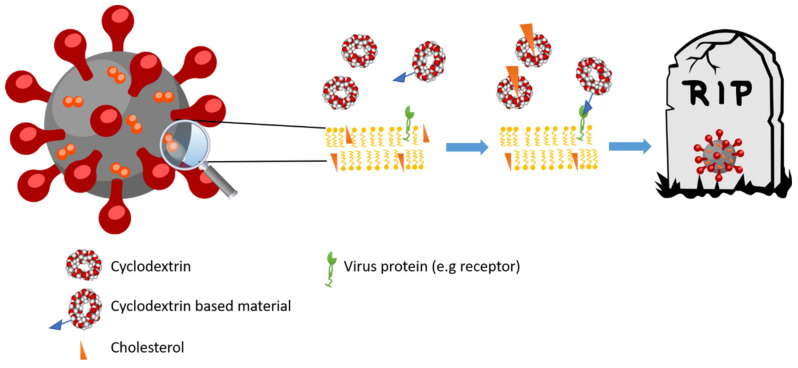
Schematic mechanism of action of CDs and proposed CD-based materials against virus. CDs are able to complex the cholesterol of the membrane, and different materials could also block other targets such as membrane receptors.

The capacity of CDs to complex cholesterol can be used to induce structural deformation in virus membranes containing cholesterol (Figure 3). Such is the case with the influenza virus in which the presence of RAMEB (randomized Mβ-CD) not only deforms the membranes [88], but also reduces the infectivity of viral particles of influenza A (H1N1 strain) in vitro [89,90]. However, these results lack the foundations for producing novel therapies against influenza virus.

On the other hand, several CD derivatives have been designed for the treatment of influenza, including a family of pentacyclic triterpene-functionalized α-, β-, and γ-CD derivatives [91,92] and fullerene–cyclodextrin conjugates [93]. The terpenic derivative was tested in vitro with promising (but limited) results showing no toxicity and good affinity for hemagglutinin. Recently, 18 water-soluble β-cyclodextrin–glycyrrhetinic acid conjugates [94] were tested against the influenza virus of which six showed promising antiviral activity. About glycyrrhetinic acid, the C-3 and C-30 were showed the best positions to modify. However, more experiments are needed to determine the real potential of these novel derivatives.

In Japan, some researchers have been evaluating the first CD-adjuvanted vaccine in history, in this case against the influenza virus [95]. The vaccine uses CDs as adjuvant because of its ability to enhance the production of antibodies (by nearly 30%) and to induce dendritic cell maturation [96,97].

#### 3.3.2. HIV

In the same way, the capacity of CDs (in this case HPβ-CD) to complex cholesterol (Figure 3) can be used to decrease the infectivity of HIV and SIV (simian immunodeficiency virus) [98,99]. This capacity was demonstrated in vivo using a mouse model in which the vaginal administration of HPβ-CD blocked 91% of the infection [100]. However, the results in rhesus macaque advised against the use of HPβ-CD in repeated doses: while HPβ-CD blocked the first infection, the prolonged treatment with HPβ-CD (when the viral inoculation was repeated 11 or 47 weeks later) increased the infectivity causing a large-scale infection [101]. These data point to the need for further research before this treatment can be recommended for use against HIV virus. On the other hand, the treatment of monocytes cultured with HPβ-CD was seen to reduce inflammatory molecules such as interleukins (IL-10) and cytokines (TNF-α, tumor necrosis factor alpha) [102]. Finally, novel-branched CD bearing long-chain alkyl group have been developed to penetrate and be fixed into the lipid bilayer of the HIV virus, while sulfated maltoheptaose moieties have been found to electrostatically interact with HIV gp120 molecule and so be used for anti-HIV applications [103,104].

#### 3.3.3. Coronavirus

The SARS-CoV-2 pandemic and its impact on society demands new therapies to prevent infection and manage the disease. In this case, it is possible that CDs (Figure 3) could be used as agents to prevent and treat infection not only by complexing the approved drugs, but also as a drug *per se* [105]: the use of Mβ-CD was previously reported to lessen the infectivity of coronavirus infectious bronchitis virus (IBV) [106], or by affecting the lipid rafts and the levels of angiotensin-2 [107]. In addition, different derivatives of CD-based materials may have the capacity to block coronavirus [108]. More information about different mechanisms of CDs against SARS-CoV-2 (e.g., in inclusion complexes containing drugs), and also as active drugs, is available in this review [109].

#### 3.3.4. Other Viruses

It is clear that cholesterol-dependent viruses can be affected by CDs (Figure 3). For example, the capacity to reduce the infectivity of dengue virus was suggested to be due to the presence of cholesterol in membrane [110]. In this respect, CDs were seen to be able reduce the infectivity of this virus in a monocyte cell model (U937 myelomonocyte cell line) [111], and the same was true in the case of Herpes Virus 1 [112], varicella-zoster virus (VZV) [113], hepatitis C virus [114], or Enterovirus D68 [115].

On the other hand, these viruses are also HS-dependent (generally Heparan Sulfate Proteoglycans) [116] and novel highly sulfonated CD derivatives (sodium undec-10-enesulfonate with different chain lengths) have been tested against this type of viruses (respiratory syncytial virus, human metapneumovirus, dengue virus, hepatitis C, and others) in vitro, ex vivo, and in an animal model. The derivatives exhibited a broad-spectrum virucidal, irreversible mechanism of action, and high biocompatibility and also acted as a barrier to viral resistance. Moreover, to determine the inhibition mechanism of these novel CDs, a molecular dynamic simulation was carried out in presence of glycoprotein B (gB) from HSV-2. The results suggested that the CDs interacted with the binding loop of gB, producing a conformational change in the protein, thus blocking the attack on the host cell.

### 3.4. Antiparasitic Activity

As previously reported [18], CDs also have antiparasitic CD applications: in the case of Leishmaniasis, for example, the use of HPβ-CD in BALB/c mice infected with the parasite *Leishmania donovani* caused a 21% reduction in liver infection compared with a control, due to its ability to complex cholesterol [117]. Although the use of other CDs such as Mβ-CD has also been studied [118], more research is necessary before a useful therapy is available. Another interesting application of CDs could be against malaria, which is caused by protozoa parasites of the *Plasmodium* genus; in this sense, sulfated CDs were able to block the entry of *P. falciparum* [119].

### 3.5. Anti-Anesthetic Agent

Neuromuscular blocking (NB) is used during surgery to prevent movement. Several agents can block acetylcholine in nicotinic receptors in striated muscle cells. Although there are different ways to remove the anesthetic agent, using CDs was found to be a simple way to complex the molecules. Using rocuronium bromide, one of the most widely used anesthetic molecules as a model to test the ability of natural CDs, the most suitable form for complexing was found to be γ-CD [120]. However, a derivative called sugammadex, obtained by perfunctionalization of the primary hydroxyl side of γ-CD with sulphanylpropanoic acid, has generated very stable complexes, which have been approved by the EMA and FDA for therapeutic use [121]. In addition to the capacity to complex rocuronium bromide, sugammadex is also able to complex vecuronium bromide and pancuronium bromide and needs less time to revert the anesthetic than neostigmine (the classical agent used) [122], attaining good clearance in 24 h [123]. Although sugammadex is generally safe, cases of anaphylaxis (a life-threatening clinical condition that is typically the result of drugs or substances used for anesthesia or surgery) have been reported with a low incidence of 29 per million cases [124]. Additional information about this use can be found in this review [18].

### 3.6. Dietary Function: Fiber, Prebiotic, and Fat Reduction

**Figure 4:**
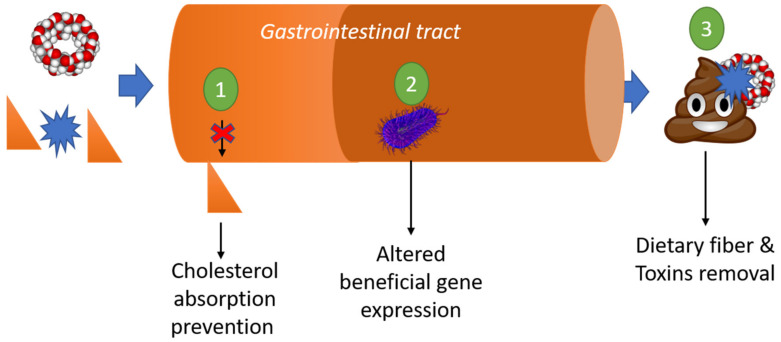
Schematic action mechanism of CDs and CD-based materials in the gastrointestinal tract.

The role of CDs as a nutritional supplement has been evaluated in patient consuming (Figure 4) cholesterol-rich diets, finding that CDs are able to reduce hypercholesterolemia by reducing cholesterol absorption, and even plasma cholesterol or triglyceride levels [125,126,127,128]. Another study found supplementation with α-CD altered the gut microbiota and increased the production of lactic acid and short-chain fatty acid (SCFAs). This had beneficial antiobesity effects by modulating the expression of genes related to lipid metabolism, indicating the prebiotic property of α-CD due to its metabolization [10,129].

Finally, the EFSA permitted α-CD to be described as dietary fiber and is suitable for reducing post-prandial glycemic responses due to its competitive inhibition of α-amylase [34].

### 3.7. Removal of Organic Toxins

The sequestering properties of CDs and CD-based materials can be exploited to remove contaminants and toxins (Figure 4). In an interesting study, insoluble β-CD beads polymers (BBP) were tested to remove Zearalenone (ZEN), a *Fusarium*-derived mycotoxin, which exerts xenoestrogenic effects in animals and humans and is formed in cereals and cereal-based products [130]. The results showed that even relatively small amounts of BBP can strongly decrease the mycotoxin content of aqueous solutions (including beer), and they can be easily recycled with an EtOH/water (50:50) solution. In another study alternariol (AOH), a mycotoxin that occurs in wine and tomato products as a contaminant was removed by BBP from aqueous solutions (pH 3.0–7.4). BBP strongly decreased the AOH content of both wine and tomato juice samples, suggesting the suitability of CD polymers as AOH binders in some beverages [131].

Moreover, a study about cyclodextrin nanosponges for removing organic toxic molecule from the body was recently published [132]. Cyclodextrin-based nanosponges are cross-linked polymer structures with a three-dimensional network tunable particle size and good swelling properties [14]. In the above article, different nanosponges were tested to complex indole, a metabolite of tryptophan formed by the gut microbiota, which can form dangerous uremic toxins, such as indoxyl sulfate, which is metabolized from indole in the liver. Three of the four nanosponges tested were able to adsorb indole from aqueous solutions as well as from simulated gastric fluid. Toluene diisocyanate cross-linked CD-nanosponges, especially, had a very high indole adsorption capacity (over 90%) and is a promising agent for cleansing the body of toxic compounds from food or through oral ingestion in general. In addition, this derivative was more stable in gastrointestinal media. Animal studies further revealed that orally administered CD-NSs do not tend to accumulate and damage gastrointestinal tissues and are excreted from the GI tract with minimal absorption [132].

### 3.8. Collagen

Recent studies have demonstrated the ability of CDs to modulate collagen-related processes: Mβ-CD was able to up-regulate collagen I expression in chronologically-aged skin through its anti-caveolin-1 activity; the intra-dermal administration of a 2.5% concentration of Mβ-CD (twice per week for two months) showed a strong collagen I up-regulation activity, leading to an increase in skin thickness without adverse reactions such as skin fibrosis [133]. In addition, CDs can interact with hydrophobic amino acid residues of collagen for different uses: the application of β-CD to collagen vitrigels produces materials with aligned fibers and lamellae similar to those of the native cornea, resulting in mechanically robust and transparent materials that can be used to create β-CD/collagen implants with a curvature matching that of the cornea. The implants show good tissue integration and support re-epithelialization [134]. Collagen–glycoseaminoglycan scaffolds that incorporate β-CD showed improved sequestration as well as the extended retention and release of TGF-β1 (transforming growth factor beta 1) and BMP-2 (bone morphogenetic protein 2), which influence the metabolic activity and proliferation of mesenchymal stem cells. Moreover, a gene expression analysis showed that the TGF-β1 released from β-CD promoted early chondrogenic-specific differentiation [135]. Finally, for the treatment or prevention of cartilage degeneration and arthrosis or arthritis a patent has been registered for the use of CDs with hyaluronate and chondroitin [136].

### 3.9. Cell Differentiation

In the last section, CDs were able to promote the differentiation of chondrocytes by complexing several molecules (such as TGF-β1). However, there are some cases where CDs interacts directly with the pathway. In 2017, a study demonstrated that β-CD could induce the differentiation of resident cardiac stem cells to cardiomyocytes through autophagy [137]: β-CD increased the expression of cardiac transcription factors and structural proteins among others to induce cardiac stem cells differentiation. In addition, JNK/STAT3 (c-Jun N-terminal kinase/signal transducer and activator of transcription 3) and GSK3β/β-catenin (glycogen synthase kinase 3 beta/β-catenin) pathways were showed as downstream pathways of β-CD-induced autophagy and differentiation. Moreover, β-CD performed its functions by improving intracellular cholesterol levels and so affecting cholesterol efflux [137].

### 3.10. Contact Lenses

The use of CDs as a carrier in liquid solutions for contact lenses is extensive, but a recent patent [138] points to the role of Dexolve™ (SBECD, a CD derivate) as a pharmaceutically active agent to prevent, treat, or reduce the risk of disorders or conditions associated with the wearing of contact lenses. CDs reduce the concentration or the bioactivity of an eye allergen, inflammatory mediators, or toxic aldehyde. CDs may, therefore, inactivate mediators of the inflammatory response, such as prostaglandins, reactive aldehydes, and lipid peroxidation products. Although this is a promising application, more studies are needed.

## 4. Cyclic Nigerosyl-1,6-Nigerose as Active Drug

In contrast to the vast literature on CDs, very few examples of the applications of CNN have been described to date, and what is known is presented below. More specifically, this paragraph deals with the use of CNN as a diet supplement and as immunological modulator.

### 4.1. Dietary Function

In a patented study, a group of rats received a diet containing 0–5% of CNN and supplemented with 3.5% of a mineral mix containing calcium, phosphorus, magnesium, iron, sodium, and potassium. A dose-related increase in the absorption rates of calcium, magnesium, phosphorus, and iron was reported [139].

Two studies demonstrated the ability of CNN to increase the production of SCAFs. In one study, a dose of 7480 mg/kg in mouse led to statistically significant increases in butyrate and lactate levels [140]. In a second study, similar results for the production of SCFAs were obtained in rats; furthermore, a decrease in serum triglyceride and cholesterol levels was observed for the high-dose diet (≈5000 mg/kg) [39].

### 4.2. Immunological Modulator

The dietary supplementation of CNN was increased the production of IgA (Immunoglobulin A) in mice, also affecting the levels of IL-6 and TGF-β (transforming growth factor beta) in Peyer’s patch cells [140]; the authors suggested a possible prebiotic effect of this molecule, which could change the microbiota profile. The increase in the production of IgA, the major antibody secreted into the gut, was studied in colitis induced in mice. The CNN-treated mice with induced colitis showed an improvement in colitis factors (e.g., mRNA levels of interleukin-1) compared with the CNN-untreated mice. Although there was no difference in the IgA concentration among groups, a higher proportion of cecal microbiota was coated with IgA in the CNN-treated group compared with that observed in the control. IgA plays a crucial role in suppressing gut inflammation due to commensal gut microbiota. The authors concluded that CNN treatment reduced gut inflammation in mice with induced colitis, possibly through synergistic effects of the restoration of goblet cells, increased abundance of butyrate-producing bacteria, and promotion of IgA coating of gut microbiota [141].

Finally, the use of CNN was effective in the treatment of melanoma in vitro. CNN administered to B16 melanoma cells resulted in a dose-dependent decrease in melanin synthesis, even under conditions that stimulate melanin synthesis, with no significant degree of cytotoxity. CNN was able to slightly reduce the tyrosinase activity directly and to moderately decrease its expression. Moreover, the colocalization of the enzyme in LAMP-1 organelles (where tyrosinase is degraded) was observed [142].

## 5. Critical Perspective of the Structure–Activity Relationship

As is clear from the previous paragraphs, cyclic oligosaccharides can play several different roles (as antiviral, PD treatment, removal of organic toxins, etc.) The principal factor that determines the activity is their affinity of the target for the internal cavity (Figure 1). In the case of CNN, we have seen how this cyclic oligosaccharide is able to increase the absorption of several ions, increasing their bioaccessibility, through the formation of complexes. CNN is able to interact directly with an enzyme, inhibiting its activity and increasing the production of IgA as a prebiotic. However, more research in this field is needed before CNN can be administered as a drug.

Most of the applications of CDs mentioned in this review are related to their ability to complex cholesterol, especially in the case of β-CD and its derivatives [143,144]. The type and degree of substitution of CD derivatives have been correlated with cholesterol complexation [144]; these authors reported that methyl-β- derivatives provided better solubilization (and complexation) of cholesterol, with an optimum degree of substitution of fourteen. Although other derivatives such as HPβ-CD presented a lower capacity to solubilize cholesterol, significant differences were found between the cytotoxicity of highly toxic derivatives of methylated compounds (IC_50_: ≈ 50 mM, except those with a low degree of substitution) and other CDs. The studied non-methylated derivatives, such as HPβ-CD and ionic β-CDs, presented no cytotoxicity up to 200 mM. Indeed, as we have shown in this review, HPβ-CD is used for diseases such as NPC due to its low toxicity. The authors of the above-cited article concluded that in the case of methylated-β-CD compounds, the cell toxicity closely depends on the number and only slightly on the position of the methyl groups [144]. In the case of sugammadex and its better ability to complex rocuronium bromide, γ-CD has the best suited inner diameter for this purpose. The addition of sulphanylpropanoic acid to the primary -OH group of γ-CD increased of the cavity depth, presenting an anionic charge, which increases the affinity for rocuronium bromide and similar [120].

On the other hand, CDs can also interact with proteins as in Alzheimer disease or Parkinson disease, because the hydrophobic amino acids of the proteins are also suitable for complexation. Indeed, CDs are able to improve the tridimensional structure of a protein acting as chaperones [145,146]. This effect also suggests the same use for other aggregates. Finally, we have described novel derivatives with structures better able to complex some molecules (see section on removing toxins) or to interact with certain receptors (see antiviral section). This ability can be used to carry a given molecule to the target [147] or to directly block the target.

## 6. Concluding Remarks

The present review emphasizes the role of CDs and CNN as active agents in themselves, rather than as carriers. Their excellent biocompatibility, good interactions with biomolecules, and the fact that they can be easily functionalized make cyclic oligosaccharides highly versatile and multitasking materials suitable for a wide set of different applications. For example, the capacity of CDs to complex cholesterol can be exploited not only to prevent diseases such as NPC or atherosclerosis, but also to prevent viral or parasitic infections. Moreover, their ability to disaggregate a variety of molecules, including proteins, suggests a possible application in the treatment of other diseases, such as Parkinson’s. They can be used as dietary supplement to control cholesterol uptake and to remove toxins. In addition, when used in combination with collagen, they present interesting properties as scaffold and as pharmaceutically active agents in contact lens solutions. CNN is able to increase the absorption of ions and the expression of IgA, thus acting as an immunological modulator.

As a final remark, it should be mentioned that several research groups worldwide are currently working to unlock the full potential of cyclic oligosaccharides and their derivatives, so that new and surprising applications can be expected in the near future.
